# Environmental and Molecular Drivers of the α-Gal Syndrome

**DOI:** 10.3389/fimmu.2019.01210

**Published:** 2019-05-31

**Authors:** Alejandro Cabezas-Cruz, Adnan Hodžić, Patricia Román-Carrasco, Lourdes Mateos-Hernández, Georg Gerhard Duscher, Deepak Kumar Sinha, Wolfgang Hemmer, Ines Swoboda, Agustín Estrada-Peña, José de la Fuente

**Affiliations:** ^1^UMR BIPAR, INRA, ANSES, Ecole Nationale Vétérinaire d'Alfort, Université Paris-Est, Maisons-Alfort, France; ^2^Department of Pathobiology, Institute of Parasitology, University of Veterinary Medicine Vienna, Vienna, Austria; ^3^Molecular Biotechnology Section, University of Applied Sciences, Vienna, Austria; ^4^Biology Center, Institute of Parasitology, Czech Academy of Sciences, Ceské Budějovice, Czechia; ^5^FAZ-Floridsdorf Allergy Center, Vienna, Austria; ^6^Faculty of Veterinary Medicine, University of Zaragoza, Zaragoza, Spain; ^7^SaBio, Instituto de Investigación de Recursos Cinegéticos, IREC-CSIC-UCLM-JCCM, Ciudad Real, Spain; ^8^Department of Veterinary Pathobiology, Center for Veterinary Health Sciences, Oklahoma State University, Stillwater, OK, United States

**Keywords:** red meat allergy, food allergy, α-Gal syndrome (AGS), ticks, IgE

## Abstract

The α-Gal syndrome (AGS) is a type of allergy characterized by an IgE antibody (Ab) response against the carbohydrate Galα1-3Galβ1-4GlcNAc-R (α-Gal), which is present in glycoproteins from tick saliva and tissues of non-catarrhine mammals. Recurrent tick bites induce high levels of anti-α-Gal IgE Abs that mediate delayed hypersensitivity to consumed red meat products in humans. This was the first evidence that tick glycoproteins play a major role in allergy development with the potential to cause fatal delayed anaphylaxis to α-Gal-containing foods and drugs and immediate anaphylaxis to tick bites. Initially, it was thought that the origin of tick-derived α-Gal was either residual blood meal mammalian glycoproteins containing α-Gal or tick gut bacteria producing this glycan. However, recently tick galactosyltransferases were shown to be involved in α-Gal synthesis with a role in tick and tick-borne pathogen life cycles. The tick-borne pathogen *Anaplasma phagocytophilum* increases the level of tick α-Gal, which potentially increases the risk of developing AGS after a bite by a pathogen-infected tick. Two mechanisms might explain the production of anti-α-Gal IgE Abs after tick bites. The first mechanism proposes that the α-Gal antigen on tick salivary proteins is presented to antigen-presenting cells and B-lymphocytes in the context of Th_2_ cell-mediated immunity induced by tick saliva. The second mechanism is based on the possibility that tick salivary prostaglandin E2 triggers Immunoglobulin class switching to anti-α-Gal IgE-producing B cells from preexisting mature B cells clones producing anti-α-Gal IgM and/or IgG. Importantly, blood group antigens influence the capacity of the immune system to produce anti-α-Gal Abs which in turn impacts individual susceptibility to AGS. The presence of blood type B reduces the capacity of the immune system to produce anti-α-Gal Abs, presumably due to tolerance to α-Gal, which is very similar in structure to blood group B antigen. Therefore, individuals with blood group B and reduced levels of anti-α-Gal Abs have lower risk to develop AGS. Specific immunity to tick α-Gal is linked to host immunity to tick bites. Basophil activation and release of histamine have been implicated in IgE-mediated acquired protective immunity to tick infestations and chronic itch. Basophil reactivity was also found to be higher in patients with AGS when compared to asymptomatic α-Gal sensitized individuals. In addition, host resistance to tick infestation is associated with resistance to tick-borne pathogen infection. Anti-α-Gal IgM and IgG Abs protect humans against vector-borne pathogens and blood group B individuals seem to be more susceptible to vector-borne diseases. The link between blood groups and anti-α-Gal immunity which in turn affects resistance to vector-borne pathogens and susceptibility to AGS, suggests a trade-off between susceptibility to AGS and protection to some infectious diseases. The understanding of the environmental and molecular drivers of the immune mechanisms involved in AGS is essential to developing tools for the diagnosis, control, and prevention of this growing health problem.

## Introduction

Most mammals express the antigen Galα1-3Galβ1-4GlcNAc-R (α-Gal). Inactivation of the α-1,3-galactosyltransferase (α1,3GalT) gene in old world monkeys, apes, and humans resulted in an almost unique ability of this group of primates to produce high antibody (Ab) titers against α-Gal (1). An α1,3GalT gene (*GGTA1*) is also present in the human genome. However, in human cells only truncated transcripts of this gene have been detected. These transcripts lack the two catalytic exons of galactosyltransferases and are thus translated in an enzymatically inactive α1,3GalT polypeptide (2). Therefore, humans cannot synthesize the α-Gal epitope. Instead, all non-immunocompromised humans can develop a strong immune response against this non-self-recognized oligosaccharide (3). Abs directed against α-Gal are regarded as the only abundantly expressed natural Abs in humans (3, 4). It has been suggested that anti-α-Gal Abs are constantly produced in the gastrointestinal tract against α-Gal epitopes present in the outer membrane of bacteria from the intestinal microbiome (3). Owing to the continuous antigen stimulation by the gut microbiome, also a large number of blood B lymphocytes have the capability to produce Abs directed against α-Gal (5–7). Most of these blood B cells are memory B cells, but once foreign antigens expressing α-Gal enter the body, these anti-α-Gal B cells are stimulated and can produce large amounts of high-affinity α-Gal Abs (7, 8). Abs raised against microbiome produced α-Gal have been described to be IgM and IgG, predominantly IgG2. Indeed, IgG Abs to carbohydrates are mostly IgG2 (9, 10).

The outcome of an immune response against foreign α-Gal epitopes can be either beneficial or detrimental, depending on the source of α-Gal and how and where the body encounters α-Gal (11). A beneficial effect of anti-α-Gal Abs is the protection against the transmission of vector-borne and non-vector-borne pathogens that also carry α-Gal on their surface (6, 12). In contrast, a negative effect of the immune response against α-Gal is the rejection of xenotransplants, caused by the recognition of α-Gal expressed on pig cells, which prevents the transplantation of pig organs into humans (13). Another detrimental effect of anti-α-Gal immunity is the α-Gal syndrome (AGS), which is caused by IgE Abs directed against α-Gal and which is characterized by two different forms of anaphylactic reactions: delayed allergic reactions after ingestion of red meat and immediate reactions in response to tick bites and during intravenous exposure to cetuximab, a chimeric mouse-human IgG1 monoclonal Ab (mAb) specific for the epidermal growth factor receptor (EGFR) used in the treatment of colorectal cancer (14–17).

In this manuscript, we reviewed our current knowledge and hypothesis on the environmental, immune and molecular factors associated with this recently discovered tick-related disease.

## Clinical Aspects of AGS

The term “syndrome” has recently been proposed to better describe the clinical relevance of this unique allergy induced by tick bites (18, 19). The distinctive clinical feature of AGS mainly relates to a delay in the onset of systemic allergic reactions in α-Gal sensitized individuals that typically occur 3–6 h after red meat consumption, which makes the AGS different from all other classical IgE-mediated food hypersensitivities (19, 20). However, immediate anaphylaxis to tick bites and cetuximab has also been reported (14–17). The mechanisms underlying the delay in response to red meat consumption are still poorly understood, but the factors involved in digestion, absorption and subsequent presentation of α-Gal molecules to the host immune system after red meat consumption appear to be very important (19, 21). Specifically, delayed appearance of glycolipid forms of α-Gal in blood circulation following the alterations in lipid and fatty acid metabolism is a plausible explanation for the delay in symptoms development (21). Moreover, in the same study Steinke et al. (21) reported significant and time-dependent differences in baseline expression of several metabolites between the healthy control group and meat allergic subjects, with the highest disparity being observed in amino acid/peptide and lipid metabolic pathways. These findings further suggest that metabolic changes including those related to amino acid catabolism, lipid, and carbohydrate metabolism and bile acids synthesis are all associated with AGS development and its presentation, likely through alterations of components involved in the allergic response.

In general, AGS occurs in patients of all ages with no obvious connection to a previous atopic disposition (22). However, there are studies that described an impact of age and atopy on the development of AGS (23–25). Affected humans display a variety of clinical symptoms and these commonly include urticaria, pruritus, recurrent angioedema, or even life-threatening signs of systemic anaphylaxis. Some individuals also described nausea, diarrhea, indigestion, or abdominal pain prior to the onset of the syndrome. Several patients with AGS report a complete disappearance of the clinical symptoms, even after new exposure to α-Gal, indicating the idiosyncratic nature of AGS (19, 20). A recent study in forest workers with high exposure to tick bites revealed sensitization against α-Gal in as many as 35% of them, but AGS was diagnosed in <5% of the sensitized subjects (25). These clinically distinct types of the syndrome presentations suggest that allergen dose and/or co-factors may affect likelihood and severity of the reaction (18, 26, 27). For instance, delayed anaphylactic reactions to α-Gal are most frequently observed after beef (53%) and pork (47%) consumption followed by ingestion of lamb (9.1%) or deer (7.3%) meat (18). Moreover, eating pork kidney and other mammalian innards often results in a more severe and rapid reaction with clinical symptoms appearing in <2 h after meat consumption (26, 28). Several studies have revealed that pork kidney contains quantitatively more α-Gal epitopes compared to muscle meat, indicating that difference in severity and temporal dynamics of the anaphylaxis in patients with AGS is closely associated with the amount of accessible α-Gal determinants in the meat (18, 26, 29).

The way in which α-Gal is processed and absorbed after ingestion of mammalian tissues is not yet well-defined (21). Apart from the concentration of allergen ingested and individual components involved in the modulation of the digestion processes, some other factors may influence the risk of α-Gal-sensitized individuals to develop an allergic reaction to red meat (18, 30, 31). Concomitant alcohol consumption, physical exercise and use of some type of medications (e.g., non-steroidal anti-inflammatory drugs, acetylsalicylic acid) are associated with variations in the clinical presentation of AGS, for instance shorter delay of reactions in patients with IgE to α-Gal (23, 26, 28, 32). The general hypothesis is that these co-factors increase gastrointestinal absorption and uptake of the allergen by for example; increasing gastrointestinal permeability, blood circulation, and histamine release (33). Therefore, AGS could be classified as food-dependent exercise-induced anaphylaxis (FDEIA) due to the striking effect of the modifying co-factors on the timing of allergic reactions to meat as well as on the presentation of α-Gal moieties to the patients' immune system (28). However, more in-depth studies are required to better understand the effects of different co-factors and mechanisms involved in the development and severity of AGS.

Aside from being involved in allergy to red meat, sensitization to α-Gal is also associated with allergic reactions in individuals exposed to different sources of α-Gal-containing antigens, other than meat and meat by-products (19, 34). Immediate onset anaphylaxis has been reported in patients with metastatic colorectal cancer treated with cetuximab. Due to intravenous infusion of the cancer drug containing high concentration of α-Gal epitopes on the FAB portion of this molecule's heavy chain, reaction to cetuximab develops immediately and may even be fatal in highly sensitized persons (15, 35). Administration of vaccine and drugs containing mammalian products such as gelatin, collagen or albumins, may also confer a risk of the allergic reaction in individuals with anti-α-Gal IgE Abs (34). Furthermore, results of recent studies have shown that increased levels of IgE Abs to α-Gal have been associated with premature degeneration of porcine aortic valves (36) and increased atheroma burden and plaques, which represents a potentially new risk factor for coronary atherosclerosis (37). Finally, the AGS has been also associated with anaphylactic reactions to tick bites, and the mechanisms associated with these reactions have not been characterized but likely include modulation of the immune response by tick-derived molecules inoculated into the host during blood feeding (17, 32).

## AGS Diagnosis and Prevention

Diagnosis of AGS differs from that of typical food allergies because of the delay in symptoms onset that usually occurs a few hours after mammalian meat consumption. However, the time of the symptoms appearance largely depends on the allergen source (innards have higher potency compared to muscle meat) and several modifying co-factors (e.g., alcohol, exercise), which shorten the time before reactions (18, 32). The unspecific presentations of AGS including allergic reactions to tick bites make the diagnosis very complex and challenging, therefore an extensive patient's history including all clinical aspects must be taken into account before the laboratory tests are performed. Currently, confirmation of the initial diagnosis involves skin prick tests (SPT), determination of serum specific IgE Abs and food challenges (38). Exposure of a patient's skin to α-Gal-containing extracts is a widely used diagnostic approach, but high variations in sensitivity of the skin tests have been reported. In particular, SPT using commercial meat extracts is found to be unreliable as it generally yields poor or false negative results and consequently leads to incorrect guidance for patients (39). Also, SPT with native meat and meat products (prick-to-prick test) usually yields false-negative or just weak skin reactions. Injection of fresh pork or beef kidney preparations underneath the skin (intradermal test) proved to be more sensitive when compared to cooked or raw muscle meat from the same animal species (28, 39), although this is not a feasible method to be used in routine allergy diagnosis. Intradermal testing with 4% gelatin-derived colloid (Gelafundin) provides an alternative test with comparable sensitivity to skin test performed with fresh meat products (40).

Commercially available assays for quantitative measurement of serum IgE to α-Gal [threshold level >0.10 kUA/L, (31)] still represent the most reliable diagnostic tool (20), but the diagnostic value of the tests exclusively depends on the binding capacity of antigen-specific IgE Abs (41). Determination of IgE levels by using the abundantly α-Gal-decorated bovine thyroglobulin showed to be more useful for diagnosis of AGS (100% sensitivity and 92.3% specificity) in comparison to other IgE tests such as α-Gal-biotin, beef, or pork (41). However, none of these IgE tests can distinguish between individuals with AGS and those with asymptomatic α-Gal sensitization (38). Therefore, the basophil activation test (BAT) has been proposed as an additional *in vitro* diagnostic test, which may help to partially overcome this limitation (38), although this test will be limited to few specialized laboratories.

The food challenge is still considered as the gold standard in food allergy diagnosis, but using this method in diagnosis of the AGS is not recommended because of the delayed nature of the reaction and also because it may cause a severe and potentially fatal anaphylactic reaction (19). Complete avoidance of mammalian meat, meat by-products, and other α-Gal-containing foods is the only strategy proposed for preventing recurrent episodes of allergic reactions in patients (20). Although there is no strong evidence, many patients with α-Gal allergy seem to be able to outgrow the hypersensitivity and tolerate mammalian meat again either through prolonged tick bite avoidance (1–2 years) or through continued exposure to very low doses of α-Gal (19, 20, 42).

## Epidemiology of AGS

The first report of the capacity of ticks to induce red meat allergy was in 2007 when van Nunen observed allergic reactions after red meat consumption in 23 out of 25 patients, of which 24 reported at least one tick bite (43, 44). Due to its recent discovery and dissimilarities from most known food allergies, the reports on the occurrence and distribution of AGS are strongly influenced by the effort of individual research groups and clinicians. To the best of our knowledge, no country has implemented a national program to diagnose the AGS. In consequence, AGS is currently underappreciated and underdiagnosed. AGS has been reported in Australia, North America, Central and South America, Europe, Asia and Africa (Table 1). Distribution of ticks expressing α-Gal and human activities that favor exposure to tick bites seems to shape the incidence of this disease worldwide. So far, tick bites and therefore tick distribution and factors contributing to tick bite exposure are the most relevant epidemiological elements associated with AGS.

**Table 1:** Reports of AGS cases worldwide.

**Country**	**Number of cases**	Tick implicated^*^	**References**
USA	5011	*Amblyomma americanum*	(45–51)
Australia	801	*Ixodes holocyclus* *Ixodes (Endopalpiger) australiensis*	(44, 52)
Sweden	95	*Ixodes ricinus*	(53–55)
Japan	85	*Haemaphysalis longicornis* *Amblyomma testudinarium*	(31, 56–59)
Germany	56	*Ixodes ricinus*	(44, 60–62)
Spain	50	*Rhipicephalus bursa*	(17, 32, 63)
Italy	27	*Ixodes ricinus*	(31, 64–66)
France	24		(26, 44, 67–70)
Korea	>12^**^	*Haemaphysalis longicornis*	(44, 71, 72)
Switzerland	6	*Ixodes ricinus*	(73, 74)
Costa Rica	5	*Amblyomma cajennense*	(44)
South Africa	5		(44, 75)
Panama	4	*Amblyomma cajennense*	(31, 76)
Zimbabwe	1		(44)
Brazil	1	*Amblyomma sculptum*	(31, 77)
Ivory Coast	1	*Amblyomma variegatum*	(31, 78)
Norway	1	*Ixodes ricinus*	(31, 79)
United Kingdom	1		(31, 80)
Netherlands	1		(81)

* *A definitive association between these tick species and AGS has not been experimentally proven in all these reports [see van Nunen (31)]*.

** *The study by Sim et al. (72) reported 11 cases of AGS, Lee et al. (71) reported 1 case of AGS and van Nunen (44) reported that several more cases of this disease have occurred in Korea*.

## Risk Factors Associated to AGS

The knowledge of risk factors influencing the development of AGS is still very limited, but important progress has been made recently. In this regard, there is an important distinction to be made, AGS patients have high anti-α-Gal IgE Ab levels (43, 82, 83), but not all patients with high anti-α-Gal IgE Ab levels develop AGS (25, 53, 74). Two major groups of risk factors can be then distinguished: (i) those related with social activities (e.g., employment as forest worker and hunting activities) and ecological conditions (e.g., ground temperature and relative humidity) that favor exposure to ticks and tick bites, and (ii) others (e.g., blood type) that contribute to the capacity of individuals to develop strong anti-α-Gal IgE response after tick bites and therefore increase the probability to develop AGS. Currently, it is not known whether these individual risk factors are associated to specific genetic traits. In addition, as discussed above, other factors including alcohol consumption, physical exercise and use of some medications can affect the clinical outcome of AGS. It is worth mentioning that no correlation was found between age and sensitization to α-Gal in German, Italian, and Spanish cohorts, whereas in a Danish group, older individuals were found to be at higher risk to develop α-Gal sensitization (23–25).

### Ecological Risk Factors

It is currently accepted that α-Gal sensitization is induced by tick bites and susceptible individuals develop AGS (23–25, 44, 53). The relevance of tick bites to develop AGS is further supported by the fact that some AGS patients were able to tolerate the consumption of mammalian meat again after preventing further tick bites (42). In consequence, the distribution and abundance of ticks is a key factor to consider when analyzing risk factors associated with AGS (31, 43, 44, 53, 82). In fact, one of the key clues on the causal role of tick bites in the anaphylactic reactions induced by intravenous infusions of cetuximab in southeastern United States was the overlap between the region were cetuximab sensitivity and red meat allergy were reported and the distribution of the lone star tick *Amblyomma americanum* (15, 84, 85). An adequate knowledge of the distribution of ticks is necessary to evaluate the risk they pose to human health (86). In addition to *A. americanum*, other tick species (e.g., *Ixodes holocyclus, Ixodes ricinus*, and *Amblyomma sculptum*, see also Table 1) have been associated with α-Gal sensitization and AGS in different geographical regions (31). The distribution of some of these tick species is rather big and overdraws countries and even continents. Therefore, the sole abundance of ticks in a given area is not sufficient to predict the risk of α-Gal sensitization and AGS. A second important factor is the actual exposure to tick bites.

As ectothermic organisms, the life cycle of ticks is deeply influenced by the temperature. Ticks are sensitive to changes in the ground temperature and relative humidity, which drive the length of the life cycle and the mortality of the questing and molting stages (87). The tolerated environmental thresholds are species-specific: while a few tick species are plastic enough to colonize a wide range of weather conditions, most others will endure under narrower margins (88). The distribution of ticks is influenced by several factors including vegetation, climate, and habitat suitability for the main hosts of the tick (86). In exophilic ticks (those that quest in the vegetation and do not live inside the shelters of the hosts) the changes in climate are expected to exert a deep influence, which has already been empirically observed (89–93) or modeled (86, 94–96). The general pattern of spread of the investigated ticks so far is an increase in both latitude and altitude, matching the general trend to warmer autumn and winter. The changing patterns of rainfall do not influence the observed spread, probably because rain has little influence on tick survival, relative humidity or saturation deficit are the actual drivers of the tick's mortality (97). This is of particular interest for ticks that are known to be involved in AGS such as *A. americanum*. Springer et al. (98) built a published summary of collection records of *A. americanum* in USA and applied present day and future climate scenarios to forecast the range change of the tick. Future ensemble predictions for 2061–2080 forecasted minimum changes in the western range limit of the tick, with a northward expansion of suitable climate into the Upper Midwest and Western Pennsylvania. Results also pointed to a range contraction along parts of the Gulf coast and the lower Mississippi River valley.

In any case, patterns of distribution of ticks are also affected by the small-scale availability of adequate patches of vegetation used by suitable hosts for the tick, the tick itself, and frequently visited by humans. This might explain why α-Gal sensitization and AGS is more frequently reported in individuals from rural areas compared to individuals from urban areas (24, 32). Additionally, forest wardens, lumbermen and hunters, people with frequent contact to ticks, are also commonly affected by this type of allergy (23–25, 32). Consequently, German individuals from rural areas or in forest-related jobs have 2.48 times higher risks of developing α-Gal sensitization than the control group of a residential population (24, 25). Similarly, it was found that Danish male individuals with high tick exposure due to occupational and hobbies reasons had a higher risk to have increased levels of anti-α-Gal IgE Abs (23, 24). However, an association between gender and anti-α-Gal IgE Abs could not be observed in other cohorts from Germany and Spain (23, 25, 32), which pose questions concerning the importance of gender differences in the incidence of AGS. Alternatively, risk factors associated to gender may be relevant only in the context of some countries with specific alimentary habits or other factors yet to be identified and characterized.

Some individuals with α-Gal sensitization and AGS, however, do not recall having any recent tick bite (24). An explanation for this finding might be that these individuals did not notice the tick bite (24). The number of tick bites was also found to influence the occurrence of α-Gal sensitization and AGS (24). However, another study found no effect of the number of tick bites during 1 year on the occurrence of α-Gal sensitization and AGS (25). In contrast, the absence of tick bites reduced the anti-α-Gal IgE levels in α-Gal-sensitized individuals suggesting that tick seasonality might influence the frequency of occurrence of AGS (84). The idea of AGS seasonality was supported by the fact that the anti-α-Gal IgE levels decreased over the winter (when the questing activity of ticks is very low) in a cohort of forest service employees in Germany (25).

### Individual Risk Factors

#### Atopy

Atopic allergy is type I hypersensitivity that occurs in individuals with intense IgE-mediated immune responses after exposure to allergens such as mites, dander, food, or other substances that are otherwise innocuous. Some studies reported an association between atopy (diagnosed by SPT) and anti-α-Gal IgE positivity (23–25). The increase in anti-α-Gal IgE levels correlates with that of the total IgE and therefore atopy was postulated as an important predisposing factor to develop AGS (25). In another study, however, no correlation was found between AGS and a previous atopic disposition (22).

#### ABO Blood Groups

The structure of the α-Gal epitope is similar to that of the blood group B antigen of the ABO blood group system (10, 83). The only difference between the blood group B and the α-Gal epitope is that the former has a fucose residue, absent in α-Gal. Individuals of blood groups AB and B produce the type B antigen in their red blood cells (RBC), whereas the RBCs of individuals of blood groups A and O lack this antigen. Several studies reported that individuals with blood groups AB and B are significantly underrepresented among AGS patients (10, 41, 53, 83). In addition, individuals of blood groups AB and B produce less anti-α-Gal IgE Abs than those produced by individuals lacking antigen B in their blood groups with implications for the development of AGS (10, 41, 53). In fact, a recent report shows that type B antigen confers protection against the development of AGS (41). One study, however, did not find a significant association between different ABO blood groups and the anti-α-Gal IgE levels (25). It is worth mentioning that in the study by Fischer et al. (25) sera and not RBCs were used to identify the blood group of the patients. Identification of blood groups using sera is not very precise. One hypothesis explaining the protective role of type B antigen in AGS is that individuals of blood groups AB and B are tolerant to this antigen and do not develop a strong immunity against the self-type B antigen and the related antigen α-Gal (99). In agreement with this hypothesis, low levels of anti-antigen B Abs in blood group B individuals were associated with low levels of anti-α-Gal IgE Abs (10). Despite strong evidence of the protective role of blood groups AB and B in the development of AGS, the blood type is frequently overlooked in epidemiological studies of AGS (100). Evidence suggests that individuals of blood groups A and O may have higher risk to develop AGS compared to individuals with blood groups AB and B.

### Other Risk Factors

The α-Gal epitope can be also found on cat IgA, IgM and cat dander (23, 101, 102). Therefore, the possible association between cat IgA, IgM, cat dander and α-Gal sensitization in cat owners has been studied (23, 25). The possible association between pet ownership, especially cats, and α-Gal sensitization and AGS has not yet been fully elucidated. For example, while some groups did not find any significant AGS risk related to cat ownership (25), others observed a high correlation between cat ownership and increased levels of anti-α-Gal IgE (23). An airborne-triggered α-Gal sensitization due to cat dander was ruled out (23, 74). In addition, anti-α-Gal IgE positivity was not linked to cat allergy analyzed by SPT. Therefore, the cause of the association of cat ownership and elevated specific anti-α-Gal IgE remains obscure and possible explanations include cat scratches as a route of sensitization or introduction of ticks to human settlements by the cats. Additionally, some pet-associated endoparasites (e.g., *Toxocara* spp.) were proposed to potentially induce, beside tick bites, α-Gal sensitization in pet owners (23, 103). Although the role of intestinal roundworms of pets spilling over to humans could not be confirmed by using serum antibodies against the helminths in a Spanish cohort, the capacity of intestinal roundworms to induce α-Gal sensitization in human should be considered in future studies (23).

## Origin of Tick α-GAL

It is still a matter of debate how tick bites initiate the anti-α-Gal IgE response, whether the response is triggered by tick-derived α-Gal present in tick salivary proteins, or by mammalian glycoproteins or glycolipids that remained in the tick from a previous blood meal or by α-Gal expressed by other organisms, such as protozoan parasites, bacteria or viruses, that are transmitted by ticks. However, recent studies indicated that the anti-α-Gal IgE response is most likely caused by tick-derived α-Gal. Firstly, Hamsten et al. (54) visualized in immunolocalization experiments the α-Gal epitope in the gastrointestinal tract of *I. ricinus* ticks. Then, Araujo et al. (104) provided evidence for the presence of this epitope in the saliva of *A. sculptum* ticks by ELISA and immunoblotting (104). These authors further observed the capacity of the saliva-derived α-Gal epitope to induce an anti-α-Gal IgE Ab response in α-galactosyltransferase knockout mice either by injection of tick saliva or by tick bites (104). In another study, Mateos-Hernández et al. (17) showed the presence of tick proteins containing the α-Gal modification in *Rhipicephalus microplus* BME/CTVM23 cells and *Hyalomma marginatum* salivary glands. The molecular basis of endogenous synthesis of α-Gal in ticks was then demonstrated by the identification of three α-galactosyltransferase genes in the genome of the black-legged tick *Ixodes scapularis* (105). Heterologous gene expression in α-Gal-negative cells and gene knockdown in ticks showed that these genes are indeed involved in α-Gal synthesis and that they are essential for tick feeding and play an important role in tick-pathogen interactions (105). N-linked glycan analysis and immunolocalization confirmed the presence of α-Gal in the salivary secretory vesicles of *A. americanum* and *I. scapularis* fed with human blood, which lacks α-Gal. Furthermore, salivary samples from these ticks were able to activate basophils primed with plasma from α-Gal allergic patients (106).

All these findings indicate that tick-derived α-Gal triggers the development of α-Gal allergy. The tick-borne pathogen *Anaplasma phagocytophilum* increased the levels of α-Gal in infected tick cells suggesting that tick-borne pathogen infection may increase α-Gal levels in ticks *in vivo* (105). This finding has influenced our current way of thinking about tick-pathogen interaction since the consequences of tick infestation is not only related to transmission of pathogens to animals and/or humans, but also the increased ability of infected ticks to induce AGS in humans.

## Immunity to Tick Bites and AGS

The pathomechanism of the AGS is poorly understood. Still the question remains, why and how the transmission of the α-Gal epitope during a tick bite can induce an α-Gal-specific IgE response and in which way defense mechanisms initiated in the body contribute to the development of this response. The tick-host interface is characterized by complex interactions between the host and the arthropod. As soon as mouthparts of the tick first disrupt the epidermis and then enter the dermis, host immune mechanisms are initiated in the skin. The injury caused by the intrusion of tick mouthparts into the skin triggers in the host hemostatic responses, such as coagulation, vasoconstriction, and platelet aggregation (107). Besides, also humoral and cellular components of the innate immune system are activated such as the complement system, keratinocytes, endothelial cells, and different leukocytes, which release anti-microbial peptides and pro-inflammatory cytokines and chemokines leading to the recruitment of neutrophils and other inflammatory cells (108). Subsequently, components of the adaptive immune system contribute to the inflammatory response against ticks such as memory T and B cells that are activated and release specific cytokines or produce Abs against tick antigens (107).

Mouthparts of the tick enter the dermis after disrupting the epidermis. In the process of wound healing, a subtype of macrophages, the M2 macrophages, is involved (109). M2 polarized macrophages have the ability to suppress inflammation via upregulation of anti-inflammatory cytokines, such as interleukin (IL) 10 or transforming growth factor beta (TGF-β) to protect the host from detrimental effects of an excessive Th1 response. Additionally, tick saliva has the capacity of inhibiting the production of proinflammatory cytokines such as IL-1 in macrophages (108). In this way, tick saliva might also boost the effect of M2 macrophages. Inhibition of a Th1 immune response would in turn, skew the immune response toward Th2.

Mice successively infested by ticks showed augmented levels of TGF-β together with gradually increasing levels of IL-10 and IL-4 after every exposure to ticks (110). This finding suggests that by upregulation of Th2 cytokines, tick bites skew the polarization of the immune response toward a more anti-inflammatory Th2 cell profile that suppresses a Th1 response (108). However, Th2 immune responses induce the production of Abs of the IgE isotype and could therefore participate in the development of AGS.

Looking at the tick's perspective, the defense responses of the host can cause pain, itch, blood flow disruption in the feeding cavity, or direct damage to the tick (107), and are consequently a hindrance for effective blood feeding. Therefore, ticks developed multiple evasion strategies to counteract them. Tick saliva contains a highly complex mixture of immunomodulatory substances (108), which inhibit the mechanisms of hemostasis and suppress innate and adaptive host immune responses. Ticks differentially produce some of these substances during blood feeding (111). Compounds present in the tick saliva are able to reduce the production of pro-inflammatory cytokines such as IL-12, IL-1β or Tumor necrosis factor alpha (TNF-α) (107), and at the same time increase the production of anti-inflammatory mediators like TGF-β or IL-10 (110) that might also contribute to the generation of a Th2 response.

Prostaglandins are among the most abundant bioactive molecules in tick saliva (112). Prostaglandin E2 (PGE_2_), present in high concentrations in tick saliva, induces vasodilation, and reduces inflammation (112). PGE_2_ impairs wound healing by reducing fibroblast migration, while mediating an increased migration of macrophages, which in turn are induced to secrete more PGE_2_ (113). The hallmark of allergic diseases is the production of IgE Abs. It has been shown that PGE_2_ induces class switch recombination on B cells, leading to the production *in vivo* of IgE Abs (114). It can thus be speculated that tick salivary PGE_2_ might also stimulate a class switch to IgE Abs in anti-α-Gal B cells. In this context, it is interesting to note that defense mechanisms against parasites such as helminths are characterized by Th2 responses with elevated levels of IgE Abs that seem to have a protective function in these infections (115). However, it has been hypothesized that IgE Abs might be important for immune responses to environmental toxins such as venoms (116) and in fact, toxins are present in the saliva from a number of different tick species (117, 118). In patients suffering from AGS high levels of specific anti-α-Gal IgE Abs are accompanied by high levels of α-Gal-specific IgG1 Abs. Elevated IgG1 Abs have been observed to confer immune resistance to cutaneous feeding by ticks in guinea pigs (119). However, IgG1 Abs did not seem to have IgE-blocking activity and could not prevent anaphylactic reactions (120).

In summary, tick bites might induce allergic reactions to proteins present in the tick's saliva. In fact, anaphylactic reactions to tick proteins have been reported in several regions including Australia (44, 121) and Europe (122), among others. However, it is not yet known which exact mechanisms are behind the production of anti-α-Gal IgE Abs. Two hypotheses were proposed (99). The first hypothesis suggests that in the context of Th2 cell-mediated immunity induced by tick saliva, α-Gal expressed on tick saliva proteins is presented to antigen-presenting cells (APCs) and B cells, which would trigger the differentiation of B cells into plasma cells producing anti-α-Gal IgE Abs. The second hypothesis suggests that tick saliva contains factors, like PGE_2_, that could induce class switch recombination of pre-existing B cell clones producing anti-α-Gal IgM and/or IgG to produce IgE.

## Role of Human Immune Cells in AGS

Dendritic cells (DCs) represent a major link between the innate and the adaptive immune system since they have typical innate immune receptors, but they are also able to function as antigen presenting cells (APCs) for activation of adaptive immune responses. In the skin and in mucosal tissues, immature DCs recognize and phagocytose antigens. Owing to a concomitant activation of pattern recognition receptors, DCs mature and migrate to the draining regional lymph nodes. There they present the processed antigens in the cleft of major histocompatibility complex (MHC)I or MHCII molecules to T cells, which then initiate an adaptive immune response (123).

The way of internalization and processing of antigens determines whether an antigen is considered as harmless or as dangerous by the body. Ristivojević et al. (124) showed in *in vitro* experiments in immature monocyte-derived dendritic cells (iMDDCs) that the uptake of bovine serum albumin (BSA) increased substantially when the protein was glycosylated and carried α-Gal moieties on the surface. Furthermore, the presence of α-Gal also affected the degradation pathway of BSA since α-Gal carrying BSA was slower degraded than BSA without α-Gal (124). This suggests that α-Gal, but not the protein carrying the oligosaccharide, is recognized by the DCs as an antigen and affects the uptake and processing of the protein. The fact that it has been shown that bioactive components such as PGE_2_ present in the tick saliva can polarize the cytokine production of DCs toward a Th2 phenotype (125) might suggest that the IgE response against α-Gal is caused solely by the interaction between DCs and components of the tick saliva. However, since it is known that DCs do not produce cytokines essential for Th2 cell differentiation (such as IL-4), the interplay between the tick saliva and the host immune system apparently requires other factors for the induction of an anti-α-Gal Th2 response (126).

Mast cells play a central role in the pathogenesis of allergic diseases. They are tissue-resident granulocytes and express FcεRI, a high-affinity Fc receptor specific for the ε heavy chains of IgE Abs. Upon activation by cross-linking of FcεRI-bound IgE Abs, mast cells degranulate and release various mediators including enzymes like tryptase or chymase, cytokines also including the Th2 cytokines IL-4 and IL-13 (127), and biogenic amines such as histamine that cause the typical symptoms of allergic diseases. In contrast, the relevance of mast cells in the immune response to ticks is still not understood. Mast cell numbers do not change after a primary tick infestation but the number of mast cells rises after subsequent tick infestations (128). This might explain why IL−4 levels in mice were found to increase only after repeated infestations by ticks (110). Furthermore, ticks can either promote the secretion of histamine via a histamine release factor (129) or counteract the effects of secreted histamine by producing histamine-binding lipocalins (130, 131). These findings point to the ambiguous role of mast cells in the interplay between ticks and the host's immune system. So far studies investigating the importance of mast cells in the development of the α-Gal syndrome are missing.

Basophils are granulocytes circulating in the blood. Like mast cells, they also express the high-affinity IgE receptor FcεRI, degranulate upon activation and release histamine, and other mediators. Basophils play an important role in chronic allergic inflammation and in protective immunity against parasites (126). It is known that in the immune response against ticks, basophils are recruited to the tick-feeding site during a second tick infestation, to accumulate in the skin and play an important role as tick rejection factors (132, 133). In guinea pigs, resistance to tick infestation was associated with basophils infiltration at the tick feeding site and IgG1 Abs appeared to play a role in the basophil response to ticks (119). Patients with AGS show, together with IgE Abs to α-Gal, higher titers of IgG1 to this oligosaccharide than healthy individuals (120). Remarkably, basophils might also be involved in the initiation of Th2 immune responses since it has been shown in mouse models that they can act as non-professional antigen presenting cells (APC) and produce IL-4. In this way, basophils were able to induce epicutaneous Th2 sensitization to food antigens applied on skin lesions, and promote the development of IgE-mediated food allergy in mice (126, 134). It could be speculated that in a similar way basophils could eventually also participate in the initiation of the allergic response against α-Gal. There the injury caused by the tick mouthparts in the skin could initiate the recruitment of basophils which by secretion of IL-4 might trigger a Th2 response against α-Gal.

## Trade-Off Between AGS and Protection to Pathogens

Ticks are haematophagous ectoparasites of vertebrates and besides causing AGS, ticks transmit a wide variety of pathogens including bacteria, viruses, protozoa, and helminths (135). In particular, Lyme disease caused by the spirochete *Borrelia burgdorferi* is the most common tick-borne disease in temperate regions of North America, Europe, and Asia, and the number of reported cases has increased in the last years (136). For example, this disease is of public health concern in France where the average incidence is 47/100 000 and in some regions it can reach 200/100 000 (137). Anti-α-Gal immunity is a good model to understand the association between allergy and vector-borne pathogen transmission. Gut bacteria induce IgM and IgG anti-α-Gal Abs that are widely produced in humans (4), and at high levels these Igs protect against malaria transmission by *Anopheles* mosquitoes (6). Furthermore, α-Gal immunization protects against Chagas disease (138) and leishmaniasis (139). The anti-α-Gal Ab response may also protect against infection by other non-vector-borne pathogens such as *Mycobacterium* spp. causing different forms of tuberculosis and mycobacteriosis (140). All pathogens producing these diseases have the α-Gal epitope exposed on their surface (6, 138–140). Evidence from our lab suggests that *B. burgdorferi* expresses α-Gal on their surface. This finding suggests that anti-α-Gal IgM and IgG may protect against several pathogens expressing α-Gal on their surface. In contrast to gut microbiota, α-Gal in tick salivary glycoproteins induces a significant increase in the levels of anti-α-Gal IgE in the human host leading, as discussed in this review, to AGS. These results suggest that while IgM and IgG to α-Gal can be protective against some pathogens, IgE α-Gal might instead promote harmful allergies.

Immunity to α-Gal provides a good model to study how Abs to α-Gal might promote allergy and/or protection against pathogen infection and transmission by vectors. Evidence suggests that individuals with blood type B produce fewer anti-α-Gal IgE Abs, and that AGS is strongly associated with blood type B negative individuals (10, 53, 141). The reduced capacity of blood group B individuals to produce anti-α-Gal Abs is presumably due to tolerance to α-Gal, which is similar to blood group B antigen (10). In agreement with the negative effect of blood group B on anti-α-Gal immunity, it was recently discovered that the frequency of blood group B is positively correlated with the incidence of malaria and tuberculosis in endemic regions (140). Interestingly, Lyme disease patients do not develop high anti-α-Gal IgE when compared to AGS patients (53), even though both group of patients (i.e., Lyme disease and AGS) should have been equally exposed to tick bites. High anti-α-Gal IgE in AGS patients correlates with high anti-α-Gal IgG (10). An interesting hypothesis emerges: AGS patients who are blood group B negative may produce high levels of anti-α-Gal IgG and IgM which may protect them from Lyme disease and other diseases caused by α-Gal-containing pathogens. Hence, a strong immune response to α-Gal may protect against these diseases with the trade-off of developing AGS (11). Despite these observations, the relation between AGS and pathogen infection/transmission has never been experimentally tested. Understanding the balance between these two immune responses to α-Gal may lead to interventions to control both AGS and infectious diseases.

## Conclusions and Future Directions

The AGS is a recently reported disease present in many parts of the world and associated with tick infestations. The IgE Ab response against α-Gal is the triggering response leading to AGS but the molecules and immune mechanisms behind it are still to be discovered. The characterization of these molecules and mechanisms is essential to improve AGS diagnosis and to develop interventions for the prevention and control of this disease. Future research should be focused on the identification of tick proteins involved in the production of anti-α-Gal IgE Abs after a tick bite and the immune mechanisms leading to AGS. The relationship between tick species and AGS applying Koch's postulates in animal models would contribute to better understand disease cause and evaluation of epidemiological risks. Data on blood group type should be included in epidemiological studies to better evaluate the risks associated with blood type in the population. Other factors that may affect the AGS such as endoparasite infections and microbiota composition in both humans and ticks should be considered. Finally, the possibility of using the anti-α-Gal IgM and IgG Ab responses for the control of infectious diseases caused by pathogens with α-Gal on their surface should be developed and could contribute to controlling some of the most prevalent and mortal diseases in the world.

## Author Contributions

AC-C and JdlF conceived the study and drafted the manuscript. AH, PR-C, LM-H, GD, DS, WH, IS, and AE-P wrote specific parts of the review. All authors reviewed and approved the manuscript in its current form.

### Conflict of Interest Statement

The authors declare that the research was conducted in the absence of any commercial or financial relationships that could be construed as a potential conflict of interest.
